# Mechano-thermo-chromic device with supersaturated salt hydrate crystal phase change

**DOI:** 10.1126/sciadv.aav4916

**Published:** 2019-07-26

**Authors:** Hyunmin Cho, Jinhyeong Kwon, Inho Ha, Jinwook Jung, Yoonsoo Rho, Habeom Lee, Seungyong Han, Sukjoon Hong, Costas P. Grigoropoulos, Seung Hwan Ko

**Affiliations:** 1 Applied Nano and Thermal Science Lab, Department of Mechanical Engineering, Seoul National University, 1 Gwanak-ro, Gwanak-gu, Seoul, 08826, Korea.; 2 Manufacturing System R&D Group, Korea Institute of Industrial Technology (KITECH), 89 Yangdaegiro-gil, Ipjang-myon, Seobuk-gu, Cheonan, Chungcheongnam-do 31056, Korea.; 3 Laser Thermal Lab, Department of Mechanical Engineering, University of California, Berkeley, Berkeley, CA 94720, USA.; 4 School of Mechanical Engineering, Pusan National University, 2 Busandaehag-ro, 63Beon-gil, Geumjeong-gu, Busan 46241, Korea.; 5 Department of Mechanical Engineering, Ajou University, 206 Worldcupro, Yeongtong-gu, Suwon, Gyeonggi-do 16499, Korea.; 6 Department of Mechanical Engineering, Hanyang University, 55 Hanyangdaehak-ro, Sangnok-gu, Ansan, Gyeonggi-do 15588, Korea.; 7 Institute of Advanced Machinery and Design (SNU-IAMD), Seoul National University, 1 Gwanak-ro, Gwanak-gu, Seoul 08826, Korea.; 8 Institute of Engineering Research, Seoul National University, 1 Gwanak-ro, Gwanak-gu, Seoul 08826, Republic of Korea.

## Abstract

Active control of transparency/color is the key to many functional optoelectric devices. Applying an electric field to an electrochromic or liquid crystal material is the typical approach for optical property control. In contrast to the conventional electrochromic method, we developed a new concept of smart glass using new driving mechanisms (based on mechanical stimulus and thermal energy) to control optical properties. This mechano-thermo-chromic smart glass device with an integrated transparent microheater uses a sodium acetate solution, which shows a unique marked optical property change under mechanical impact (mechanochromic) and heat (thermochromic). Such mechano-thermo-chromic devices may provide a useful approach in future smart window applications that could be operated by external environment conditions.

## Teaser

We demonstrated a reversible transparency-changing smart glass that can be operated by mechanical impacts and heat.

## INTRODUCTION

Recently, technological convergence has stimulated the evolution of fields such as multifunctional smart devices (*1*–*3*). In particular, advanced convergence technology can provide additional functionalities to classical tools; eventually, reinventing or redefining them to yield revolutionary functions (*4*, *5*). Accordingly, various techniques for imparting new functionality to windows are under active development, including heatable (*6*), energy-generating (*7*), antibacterial (*8*), high scratch-resistant (*9*), and color-switchable windows. Among the techniques, the color-changeable smart window has been of great interest because of privacy protection, artistic designs, and effective energy management for buildings (*10*). To date, several types of smart windows have been explored such as suspended particle device (SPD) (*11*, *12*), polymer-dispersed liquid crystal (PDLC) (*13*–*22*), and electrochromic (*23*–*26*), photochromic (*27*–*29*), and thermochromic devices (*30*–*34*). In particular, each smart window has unique characteristics in aspect of working principle, operation conditions, and performance (table S1). For instance, SPDs, PDLCs, and electrochromic windows commonly use electric energy to control direction of polar particles, orientation of polar molecules, and electric charge transfer. While these electricity-based smart windows ensure fast color-switchable speed and high optical transmittance difference at bleached and colored states, electric energy has to be supplied to turn the device on/off (*11*, *35*). In the case of a photochromic window, it reacts with ultraviolet (UV) from sunlight and shows weak color switching during several minutes (*27*). Meanwhile, thermochromic devices require a constant input of thermal energy including reflected infrared energy during daytime or electrothermal heat from the microheater to activate and keep its color-shifting feature. High-energy consumption for phase-changeable metal oxide and low visibility for thermochromic pigments also limits their wide application (*36*). In this regard, a smart window that is based on a new working principle would be introduced to bring previously unknown applications and motivate further investigations.

Sodium acetate (CH_3_COONa) is a kind of salt hydrate, and it is broadly used in textile industry, pH buffer solutions, solar energy storage systems, and heating pads. Particularly, sodium acetate can contain water molecules within its crystal structure, eventually forming a hydrate (*37*). It also shows unique heat release characteristics during crystallization from a supersaturated solution. Sodium acetate can be fully dissolved in heated deionized water to be saturated, and it becomes supersaturated after natural cooling at room temperature. However, this supersaturated state of sodium acetate solution is unstable so that spontaneous phase change into crystalized sodium acetate trihydrate (*38*) and exothermic heat release occur when external mechanical stimulation such as strong shaking of the solution or sharp-tip impact is applied for the initial nucleation of sodium acetate crystals. Beside the heat release phenomena during phase change, sodium acetate shows a distinctive optical property change. Fully dissolved or supersaturated sodium acetate is a highly transparent solution, while crystalized sodium acetate trihydrate is an opaque milky colored crystal.

Although most sodium acetate–related researches have focused on the exothermic heat release feature by phase change, we demonstrated the possibility of applying sodium acetate to optical devices such as a mechano-thermo-chromic (MTC) smart window. As this chemical reaction is reversible, transparent supersaturated sodium acetate solution can instantly change into murky sodium acetate trihydrate crystals upon mechanical stimulus (mechanochromic), and the opaque murky sodium acetate trihydrate crystals can revert to the transparent sodium acetate solution state when heat is applied (thermochromic). Since the dissolving ratio of the sodium acetate crystal in solution substantially varies upon temperature, its solubility and transparency may be precisely adjusted by thermal control. By utilizing those unique optical property differences during the phase change of sodium acetate, in this study, we present a new concept of MTC device that changes and maintains its optical property by mechanical stimulus and heat. Compared to the traditional smart windows, the MTC device consists of the simplest components and enables the maintenance of high–optical transmittance state or opaque state without constant electrical/thermal energy. In particular, the mechanical stimulus on the MTC device can begin from a tiny point and bring a phase change for the whole large area. The phase-changeable sodium acetate is basically governed by thermal energy, which is similar to the typical thermochromic and chalcogenide materials. The chalcogenide-based optical modulators show extremely fast switching speed than that of sodium acetate (*39*, *40*). However, unlike a typical thermochromic window, the MTC device shows fast response time (*31*, *41*), broad optical switchable range (*3*, *33*), and transient thermal energy demand for phase change. Therefore, this new concept may provide a novel color-switchable feature for future smart window applications.

## RESULTS

### Operating mechanism

**Fig. 1:**
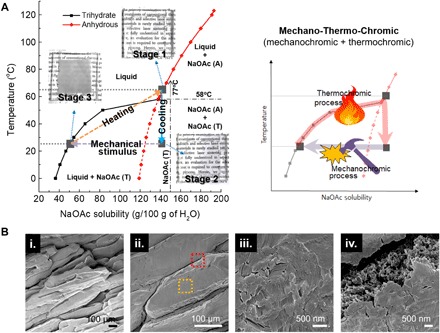
Operation mechanism and scanning electron microscopy images of MTC device. (**A**) Solubility graph at different temperature for sodium acetate anhydrous (CH_3_COONa, red line) and sodium acetate trihydrate (CH_3_COONa·3H_2_O, black line) where the solid lines indicate a saturated state. Insets show the digital pictures of MTC glass for various phase conditions. The right graph shows the general process of MTC, which is composed of the mechanochromic process and the thermochromic process. (**B**) The microscopic surface morphology of sodium acetate crystal with (i) low- and (ii) high-magnification scanning electron microscopy (SEM) images. The red dotted box exhibits the boundary of the crystal and the yellow dotted box shows the surface, which has an irregular vertically stacked structure.

The basic working principle of the deliquescent salt-based MTC device is represented in Fig. 1A. The MTC device with the integrated transparent microheater uses the sodium hydrate solution that shows three different phases with phase-dependent optical properties (transparent for the saturated/supersaturated sodium acetate solution, murky for sodium acetate trihydrate crystal) and phase change between them upon mechanical perturbation and heat. A simple chemical property such as solubility of salt in the water, i.e., water of crystallization, rules the nature of sodium acetate solution. Initially, an excessive amount of sodium acetate anhydrous (CH_3_COONa) powder dissolved in deionized water at 65°C until it reached the transparent saturated solution state (stage 1). Afterward, the sodium acetate solution naturally cooled down to room temperature; subsequently, the solution became supersaturated while still transparent (stages 1 to 2). The state of the solution changed markedly from transparent solution to opaque murky solid when the solution was subject to a mechanical stimulus (stages 2 to 3). The induced mechanical stimulus, i.e., an instant mechanical impact on the sodium acetate solution with a sharp tip, caused the supersaturated sodium acetate solution to form a sodium acetate trihydrate crystal nucleus where the crystal growth started. We studied the standard mechanical impact condition by using a spring-based impact measurement system (fig. S1, A and B, and tables S2 and S3). The result signified that a certain amount of mechanical impact energy (>40 mJ) is needed to initiate the crystallization of the sodium acetate. During the phase transition in fig. S1C, the volume change was negligible without shrinkage or expansion. While sodium acetate has two similar compounds including sodium acetate anhydrous (CH_3_COONa, *T*_m_, 324°C) and sodium acetate trihydrate (CH_3_COONa·3H_2_O, *T*_m_, 58°C), the only a noticeable difference over the compounds is found in the melting point and solubility in water. Typically, the sodium acetate anhydrous easily forms hydrates when it dissolves in water; therefore, the identity of the observed crystal in our study is sodium acetate trihydrate under 58°C. Considering the standard enthalpy for the CH_3_COONa (Δ*_f_H*^θ^_298_ = −709.32 kJ/mol) and CH_3_COONa·3H_2_O (Δ*_f_H*^θ^_298_ = −1604 kJ/mol), the major contents at stage 2 and stage 3 are CH_3_COONa·3H_2_O (*42*). This transition is reversible, and the murky sodium acetate trihydrate crystal in stage 3 could return to the clear saturated sodium acetate solution in stage 1 upon heating. This solution also cools down to room temperature in a supersaturated state by natural convection heat transfer to the environment (stage 2). Figure 1B shows the scanning electron microscopy (SEM) image of the surface morphology of the precipitated sodium acetate trihydrate crystal. The crystal has an irregular ingot form (Fig. 1B, i and ii) with a stacked lamellar structure (yellow dotted box, Fig. 1B, iii) and a spread-out ring pattern from the initial mechanical stimulus point. Moreover, many cracks (red dotted box, Fig. 1B, iv) on the surface of the sodium acetate crystal will possibly act as light scattering points to increase the optical property change.

### Designing MTC device

**Fig. 2:**
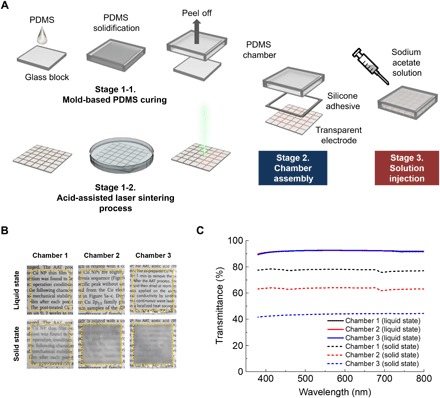
Fabrication process and optical property characterization of MTC device. (**A**) The fabrication process of MTC device that consisted of a chamber for sodium acetate solution and the transparent microheater prepared by acid-assisted laser sintering of Cu nanoparticle paste. (**B**) The digital images and (**C**) UV-vis analysis of the transparency shift after phase change for each chamber thickness (photo credit: Hyunmin Cho and Jinhyeong Kwon, Seoul National University).

The fabrication process of the MTC device is shown in Fig. 2A. The MTC device consists of three parts: a chamber, a sodium acetate solution, and a transparent microheater. We used glass blocks with 1- to 3-mm thickness and a polydimethylsiloxane (PDMS) solution to make various dimensional chambers. Highly transparent, a saturated sodium acetate solution was prepared in the syringe and kept on a hot plate until injection to the PDMS chamber to prevent unexpected crystallization. The acid-assisted laser sintering process of copper nanopaste offered a transparent microheater on the flexible polyethylene terephthalate (PET) substrate. The fabricated microheater on the flexible substrate had good electrical, chemical, and mechanical stability, which was examined in a previous study (*43*). Afterward, the PDMS chamber and the transparent microheater were bonded together for salt solution sealing and for stress accommodation during the formation of a crystal nucleus. To find an optimum optical changeable transparency property for the MTC device, we practiced simple studies on the various chamber dimensions without a transparent heater (denoted as MTC glass). As shown in Fig. 2B, the chambers with sodium acetate in its liquid state had a clear appearance because the salt solution has high transparency; it rarely hindered the overall transparency of the PDMS chamber. Accordingly, letters on a paper were visible through the MTC glass placed on the paper. The optical property dependency on the salt crystal thickness was studied for MTC glass samples with various thicknesses (chambers 1, 2, and 3 have thicknesses of 1, 2, and 3 mm, respectively). Although the transmittance of each chamber in liquid state of sodium acetate was very high, the transparency of the MTC glass gradually decreased as the thickness of the chamber increased after crystallization (Fig. 2C). Moreover, fig. S2A shows the transmittance and haze factor variation with phase change, where the haze markedly increased by more than 90% with a murky state for all the cases.

**Fig. 3:**
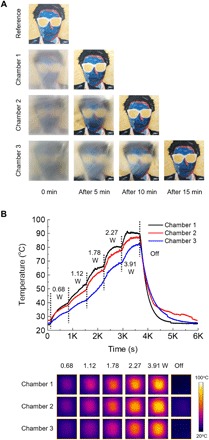
Transient thermal response characterization of the MTC device. (**A**) The optical characteristics change during the phase change of the sodium acetate crystal for each chamber at 70°C heating. The background picture used is courtesy of J.K. (**B**) The transient temperature measurement and corresponding infrared (IR) camera images for the electrothermal performance and temperature distribution of the transparent microheater depending on the thickness of each chamber.

Each state (stage 1, 2, and 3) was reversible by thermal control. The crystalized sodium acetate in the chamber can return to clear liquid state upon mild heating (stage 3 to 1). Since the heat capacity of the sodium acetate for the solid-liquid transition varied depending on the size of the chamber and corresponding material volume, the required time was also different for the same elevated temperature environment. To calculate the phase change rate difference for the crystalized sodium acetate in each chamber, we placed the MTC glass sample on a hot plate at 70°C. Transient digital camera images were taken to check the optical property change rate for the crystalized sodium acetate during heating (Fig. 3A). The top image was a reference painting image printed on a sheet of paper. Each MTC glass was directly located and overlaid on the reference image for comparison. The first-column images show a reference and initial crystalized chamber images. Chamber 1 returned to the transparent liquid state of the sodium acetate after 5 min of heating because of smaller volume of the sodium acetate solution in the chamber. Although chamber 1 has fast transition time from murky solid to transparent saturated liquid state, it has relatively lower opacity at the crystalized murky state than other thicker chambers, and the background image was still partially visible. Therefore, it cannot provide a satisfactory contrast change. In the thicker case (chamber 2), it changes into the liquid phase within 10 min. Unlike chamber 1, it provided sufficient opacity for sunlight block and security purposes. For another thicker case (chamber 3), while a light scattering property was better than that of other chambers, it needed much more thermal energy to initiate the phase transition of the crystalized salt solution with a prolonged process time of as much as 15 min. Considering that the MTC device consists of an integrated structure of sodium acetate and a transparent microheater, it is important to examine the heating performance of the integrated transparent microheater. We characterized the electrothermal performance and transparency of the laser-processed transparent microheater in fig. S2, B and C. The required electrical energy gradually increased to 0.68, 1.12, 1.78, 2.27, and 3.91 W for the all MTC devices (Fig. 3B). While the entire transparent microheater consumed the same electrical energy, we observed different heating responses depending on the chamber thickness. Since the heat capacity of chamber 1 was smaller than that of the other chambers, it showed fast responses for heating and cooling. Although the electrothermal performance of chamber 2 was slightly lower than chamber 1, it had a moderate response time and a fair temperature generation property for the MTC device. On the other hand, chamber 3 showed a slow response time and an excessive electrical energy demand for providing enough thermal energy. Compared with chambers 1 and 3, the case of chamber 2 represented a satisfactory optical property of 60% transparency from the crystalized sodium acetate and an adequate electrothermal performance from the transparent microheater of around 70°C at a 2.27-W condition; within several minutes, the MTC device was fabricated with the condition of chamber 2.

### Phase transition characteristics

**Fig. 4:**
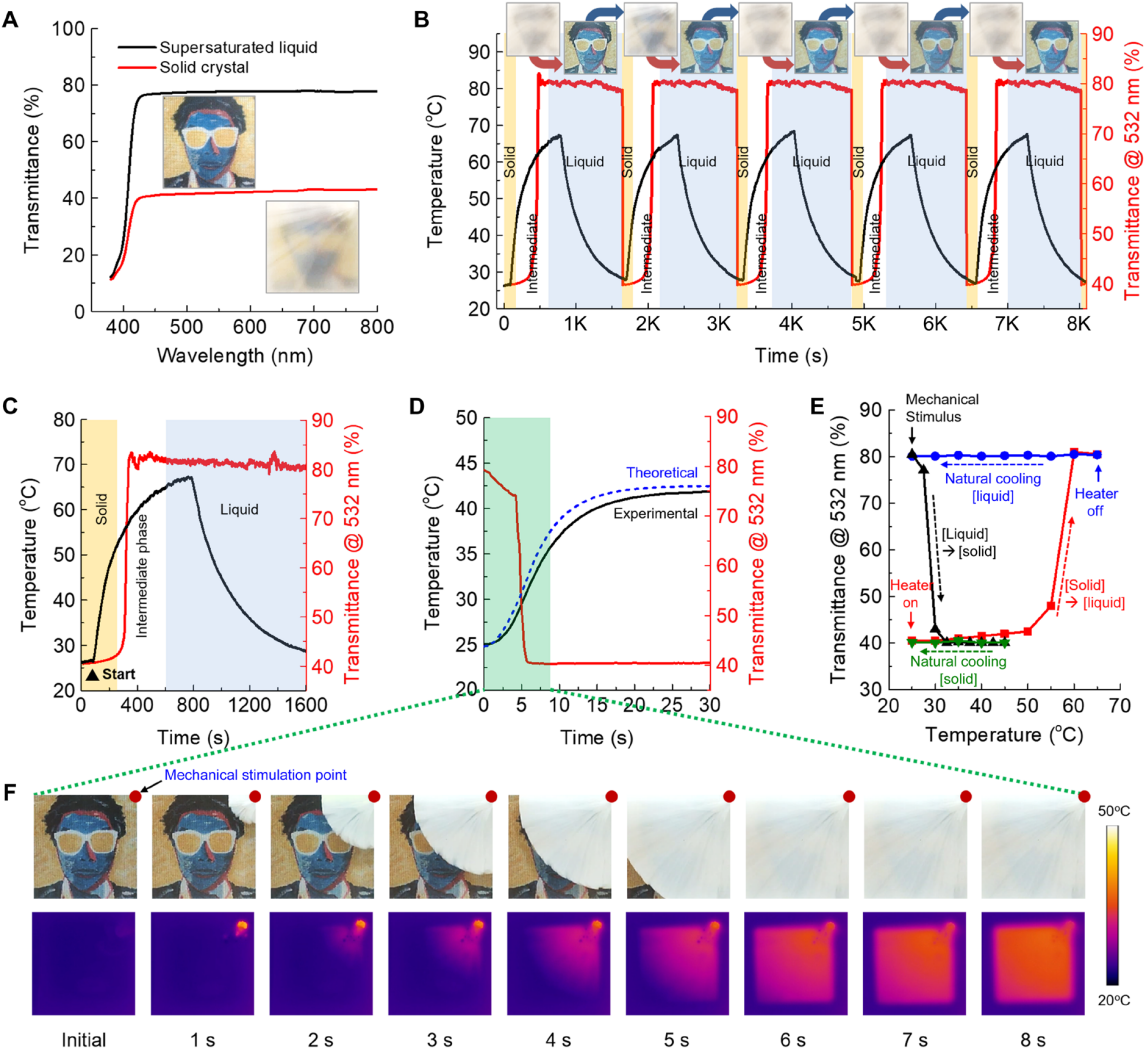
Real-time optical and thermal response during the reversible phase transition. (**A**) Spectral transmittance of MTC devices integrated with the 2-mm-thick chamber (chamber 2) and the transparent microheater depicts the supersaturated liquid phase (black line) and the crystalized solid phase (red line). (**B**) Cyclic operation test for the durability and reliability of the MTC device. Temperature (black line) and transmittance (red line) changes were recorded along with the corresponding digital images. (**C**) Real-time variation of the temperature and transmittance during the transparent microheater operation for phase change to liquid. (**D**) Real-time measurement and calculation of temperature and transmission change during the exothermic phase change of sodium acetate crystallization. (**E**) A relationship between temperature and transmittance of the MTC device during the one-cycle operation. A sharp decline (black line) of transmittance by mechanical stimulus along with exothermic reaction. Natural cooling (green line) after exothermal reaction with remaining low transparency. Phase transition to liquid (red line) by electrothermal heat from a microheater. Natural cooling (blue line) after heating with the remaining liquid state and high transmittance. (**F**) Digital picture and IR camera image during the sodium acetate crystal growth after mechanical stimulation impact at the right top corner of the sample (red dot).

The fabricated MTC device was characterized for various aspects including spectral transmittance, cyclic operation performance, transition speed, and exothermic reaction. Since the sodium acetate changes from liquid to crystal reversibly by thermal control, transparency and haze factor of the MTC device varies from 80% for the transparent liquid state to 40% for the murky crystal state (Fig. 4A and fig. S2D). The transmittance sharply dropped near the 400-nm wavelength because of the UV-curable epoxy resin in the transparent microheater that strongly absorbed the UV wavelength (fig. S2E). The reversibility of the MTC device was confirmed by a cyclic operation test (Fig. 4B), where more experiments were conducted up to 200 cycles in fig. S3A. A real-time transient transmission system with a photodetector measured the transition speed from crystal to liquid (fig. S3B). The sodium acetate shifts from the crystal to the intermediate phase and the liquid state by an external mechanical stimulation and an electrothermal heat from the transparent microheater. The MTC device showed good reliability and durability suitable for practical use. As shown in Fig. 4C, an optical visibility of the MTC device increased during the phase transition of sodium acetate from crystal to saturated liquid by the integrated transparent microheater within 400 s. The transparent microheater induced the phase change of the sodium acetate in 800 s. Since the difference of solubility at 40° and 25°C was only 14 g per 100 g of water, the precipitation amount variation for the MTC device was negligible. Therefore, the MTC device could operate again by mechanical stimulation within a few minutes. Meanwhile, the crystallization of sodium acetate was an exothermic reaction, and it released heat during the process. For this reason, we conducted experimental and theoretical investigations to show the effect of the released exothermic heat during the phase transition for the MTC device and surrounding environment as shown in Fig. 4D. The measured temperature elevation trend followed the calculated temperature (*44*). The elevated temperature was saturated around 40°C in this case, and the sodium acetate crystal did not dissolve by exothermal energy because of its solubility with water. The overall exothermic reaction occurred for 20 min (fig. S2F), and the phase transition to liquid was even possible during the exothermic reaction by heating at 65°C. A relationship between temperature and transmittance of the MTC device was presented in Fig. 4E. By controlling the phase state of sodium acetate, the MTC device showed a broad optical switchable range. Figure 4F shows the transient phase change of sodium acetate from liquid to solid, and the temperature rises due to exothermal heat release. The crystal is initiated on the spot and rapidly broadens to cover the whole area. Photocurrent variations and corresponding digital photo images confirmed that the crystal growth speed was approximately 6.56 mm/s. As shown in fig. S4, the MTC device also shows good mechanical stability against an unexpected external stimulus without direct internal mechanical impact on the sodium acetate solution. Although the impact energy (708.05 mJ), which could break the glass chamber, was applied to the MTC device more than 20 times, the sodium acetate solution at the device stayed in the liquid phase.

**Fig. 5:**
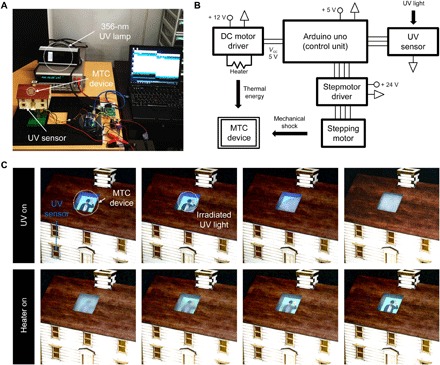
Active smart window application under UV irradiation. (**A**) A miniaturized mock-up demonstration with small MTC device–installed window and control units and (**B**) corresponding diagram. (**C**) Real-time digital images during the operation of the MTC system. The irradiation of UV light works as trigger for the sodium acetate crystallization and corresponding optical property change. The transparent microheater was turned on for phase transition from solid to liquid. See also movies S1 and S2 (photo credit: Hyunmin Cho and Jinhyeong Kwon, Seoul National University).

Smart window demonstrationAs a proof of concept for the practical application of MTC device in smart windows for a smart building system, a small MTC window and integrated control units reacting to the external stimulus enabled a simple miniaturized demonstration (Fig. 5). The mock-up system consists of a model house, a 356-nm UV lamp to simulate strong sunlight, an MTC glass window, a UV sensor, a logic circuit, and other modules. The MTC device was established on the roof of the mock-up model house, and the painting image was placed behind the MTC device to show the optical property change. A window of the house held a UV sensor. Figure S5 exhibited a logic flow chart for the MTC system operation. A mechanical stimulation module operated by a step motor generated a direct mechanical impact on the MTC system to initiate the phase transition of the sodium acetate from the liquid to the solid state. As shown in Fig. 5C, UV irradiation as a trigger launched an operation of the MTC system. When the UV sensor detected the UV light irradiation, subsequently, a stepping motor started to work to initiate the sodium acetate crystallization. Movie S1 shows the real-time operation of MTC device. The MTC device changed to an opaque state within a few seconds. Afterward, when the intensity of UV light dropped below a critical level, the microheater turned on to heat the MTC device and changed the murky sodium acetate trihydrate crystal to the transparent liquid state. The moment of sodium acetate phase change in MTC device was recorded in movie S2. The MTC system can be easily scaled up for large areas for smart window application. In contrast to the conventional electrochromic system, which needs prompt electrical energy to set the specific optical status, the optical property of MTC system could be changed by a simple mechanical perturbation, and the optical status can be maintained without external energy input. The proposed MTC device may provide a novel and simple way for energy-efficient next-generation smart windows.

## DISCUSSION

In summary, we developed a new method of active control of optical transparency for next-generation smart window application. While conventional electrochromic devices apply electric field to an electrochromic or liquid crystal material for optical property control, this study represents a new concept transparency smart window using totally new driving mechanisms (mechanical stimulus and thermal energy) to control the transparency of the salt hydrate. This developed MTC device with the integrated transparent microheater uses the sodium hydrate solution that shows a unique marked optical property change under mechanical perturbation and heat. As a proof of concept for the practical application of MTC device in smart windows for a smart building system, a small MTC window and integrated control units reacting to external environments was made possible by a simple miniaturized demonstration. The MTC device can be easily scaled up for mass production and applied in flexible substrates for nonplanar surface mounting. While the phase transition from the transparent supersaturated liquid to murky crystal is fast, the slow reverse phase transition from the solid phase to the liquid phase of the sodium acetate may be improved by adjusting the solubility of sodium acetate in water and by maximizing heat generation via a further optimized microheater design. The developed MTC devices are expected to provide a useful approach in future smart window applications that operate using an external environment condition such as intense UV light or deliberate mechanical impact from a user.

## MATERIALS AND METHODS

### Fabrication of transparent microheater

Copper nanoparticle paste sized approximately 50 nm was deposited to a grid-patterned mold, and the UV-curable epoxy resin and the doctor blade technique was conducted to transfer the Cu microgrid to the PET polymer substrate. Afterward, the Cu micropatterned electrode on a polymer substrate was immersed in buffered acetic acid solution to remove oxides and other contaminations. Last, the acid-treated Cu electrode was processed by a laser sintering process to enhance electrical conductivity and mechanical strength. The fabricated Cu electrode showed high transparent and good electrical conductivity such as 85% at 532 nm and approximately 16 ohms/sq., respectively. Detailed information on the acid-assisted laser sintering process can be found in a previous report (*43*).

### Fabrication of MTC device

Sodium acetate (140 g, 99%, anhydrous; Sigma-Aldrich) was dissolved in 100 ml of deionized water at 65°C to get a saturated solution. The sodium acetate solution cooled down to room temperature, and it formed a supersaturated solution for phase transition. The PDMS (10:1 ratio of the base and curing agent; SYLGARD 184, Dow Corning) chambers were designed for various thicknesses by different glass blocks. The PDMS chamber and the transparent microheater were glued together with PDMS adhesive (3140 RTV Coating, Dow Corning) for solution sealing. Then, the sodium acetate solution was discreetly injected into the PDMS chamber.

### Material characterization

The surface morphology of sodium acetate tryhydrate crystal was observed by SEM (JSM-7600F, JEOL). The optical property of the MTC device was characterized with UV-visible (UV-vis) spectroscopy (V-770, JASCO) for transmittance and haze measurement. The performance of the transparent electrothermal microheater and the temperature variation with sodium acetate solution solidification were investigated with an infrared (IR) camera (A645sc, FLIR), which showed the temperature and thermal distribution with time. The transmittance changes with phase transition in real time were measured by a laboratory-made system, which consisted of a laser, a beam expender, and a photodetector. The transmitted laser power varied with the state of the transition solution on a uniform area covered by the expended laser. The MTC device on the roof of the mini model house was controlled by a microcontroller board (Arduino Uno Rev3) with the control logic.

## Supplementary Material

http://advances.sciencemag.org/cgi/content/full/5/7/eaav4916/DC1

Download PDF

Movie S1

Movie S2

## SUPPLEMENTARY MATERIALS

Supplementary material for this article is available at http://advances.sciencemag.org/cgi/content/full/5/7/eaav4916/DC1 Table S1. Simple comparison of the characteristics for various smart window technologies. Table S2. Experiment conditions and results regarding mechanical stimulus and crystallization. Table S3. Digital images for the sodium acetate crystallization regarding two cases of applied mechanical stimulation. Table S4. Various factors and conditions for the calculation of exothermic heat generation. Fig. S1. Mechanical stimulus schematic, crystallization probability, and volume change for crystallization. Fig. S2. The optical and thermal behavior analysis of MTC device and microheater. Fig. S3. Cyclic durability test and real-time measure system of transmittance. Fig. S4. A pendulum impact test for mechanical external stimulus. Fig. S5. The logic flow chart for smart glass system integrated with MTC device and control units. Movie S1. The real-time operation of smart glass system by sodium acetate crystallization with mechanical perturbation when the UV sensor detected UV light. Movie S2. The real-time operation of smart glass system by sodium acetate phase change to saturated liquid state.
