# Supplementary material for: Mechano-thermo-chromic device with supersaturated salt hydrate crystal phase change
Source: Sci Adv. 2019 Jul 26;5(7):eaav4916. doi: 10.1126/sciadv.aav4916 (PMC6660208; doi:10.1126/sciadv.aav4916)
Supplement: http://advances.sciencemag.org/cgi/content/full/5/7/eaav4916/DC1 [file supp_5_7_eaav4916__index.html]

Science Advances | Science AdvancesAAASSearchScience AdvancesMenu

## Supplementary Materials

**The PDF file includes:**

- Table S1. Simple comparison of the characteristics for various smart window technologies.
- Table S2. Experiment conditions and results regarding mechanical stimulus and crystallization.
- Table S3. Digital images for the sodium acetate crystallization regarding two cases of applied mechanical stimulation.
- Table S4. Various factors and conditions for the calculation of exothermic heat generation.
- Fig. S1. Mechanical stimulus schematic, crystallization probability, and volume change for crystallization.
- Fig. S2. The optical and thermal behavior analysis of MTC device and microheater.
- Fig. S3. Cyclic durability test and real-time measure system of transmittance.
- Fig. S4. A pendulum impact test for mechanical external stimulus.
- Fig. S5. The logic flow chart for smart glass system integrated with MTC device and control units.
- Legends for movies S1 and S2

Download PDF

**Other Supplementary Material for this manuscript includes the following:**

- Movie S1 (.mp4 format). The real-time operation of smart glass system by sodium acetate crystallization with mechanical perturbation when the UV sensor detected UV light.
- Movie S2 (.mp4 format). The real-time operation of smart glass system by sodium acetate phase change to saturated liquid state.

**Files in this Data Supplement:**

- Adobe PDF - aav4916\_SM.pdf
