# Supplementary material for: Mechano-thermo-chromic device with supersaturated salt hydrate crystal phase change
Source: Sci Adv. 2019 Jul 26;5(7):eaav4916. doi: 10.1126/sciadv.aav4916 (PMC6660208; doi:10.1126/sciadv.aav4916)
Supplement: Download PDF [file aav4916_SM.pdf]

Science Advances
AAAS

advances.sciencemag.org/cgi/content/full/5/7/eaav4916/DC1

Supplementary Materials for

Mechano-thermo-chromic device with supersaturated salt hydrate crystal phase change

Hyunmin Cho, Jinhyeong Kwon, Inho Ha, Jinwook Jung, Yoonsoo Rho, Habeom Lee, Seungyong Han, Sukjoon Hong, Costas P. Grigoropoulos, Seung Hwan Ko*

*Corresponding author. Email: maxko@snu.ac.kr

Published 26 July 2019, *Sci. Adv.* **5**, eaav4916 (2019)
DOI: 10.1126/sciadv.aav4916

The PDF file includes:

Table S1. Simple comparison of the characteristics for various smart window technologies.
Table S2. Experiment conditions and results regarding mechanical stimulus and crystallization.
Table S3. Digital images for the sodium acetate crystallization regarding two cases of applied mechanical stimulation.
Table S4. Various factors and conditions for the calculation of exothermic heat generation.
Fig. S1. Mechanical stimulus schematic, crystallization probability, and volume change for crystallization.
Fig. S2. The optical and thermal behavior analysis of MTC device and microheater.
Fig. S3. Cyclic durability test and real-time measure system of transmittance.
Fig. S4. A pendulum impact test for mechanical external stimulus.
Fig. S5. The logic flow chart for smart glass system integrated with MTC device and control units.
Legends for movies S1 and S2

Other Supplementary Material for this manuscript includes the following:

(available at advances.sciencemag.org/cgi/content/full/5/7/eaav4916/DC1)

Movie S1 (.mp4). The real-time operation of smart glass system by sodium acetate crystallization with mechanical perturbation when the UV sensor detected UV light.
Movie S2 (.mp4). The real-time operation of smart glass system by sodium acetate phase change to saturated liquid state.

Table S1. Simple comparison of the characteristics for various smart window technologies.

**Index**	**SPD**	**Polymer-Dispersed Liquid Crystal**	**Electrochromic**	**Photochromic**	**Thermochromic** (Thermotropic or Thermochromic glazing)		**Chalcogenides**		**MTC Device** [This Work]
**Principle**	Polar particle orientation	Polar molecular orientation	Electric charge transfer	Photoexcitation	Phase change		Phase change		Phase change
**Material Example(s)**	Polarized particle	Nematic liquid crystal	WO3, TiO2, NiO, Polyaniline	Metal-ligand complexes, Organics	Doped-Vox	Thermochromic pigment	Ge, Sb, Te containing materials such as $Ge_2Sb_2Te_5$ or GeTe		Sodium acetate
**Operating Condition**	AC 30 – 100 V	AC 30 – 100 V	AC 1 – 3 V	UV	Heated by sunray (infrared)	Micro heater	Micro heater	Nano heater or incident light	Mechanical stimulus
					Over 70 ^{o}C*	2.5 W	2.2 V	6.5 V for crystal to amorphous 1.25 V for recrystallization	1.78 W*

**Response Time**	100 msec	10 msec	5 – 30 sec	10 min	5 min to response[#]<br>More than half hour[†]	15 sec to response<br>15 sec to recovery	MHz range	Several μS	10 sec to response<br>10 min to full recovery*
**Transmittance at 550 nm (%)**	5 – 70	60 – 80	5 – 70	60 – 80	30 – 80	10 – 50	20 – 30*	N/A*	40 – 85
**Durability**	Moderate	Weak	Good	Moderate	Good	Moderate	Good	N/A	Good
**Pros and Cons**	- Wide range of transmittance change<br>- Activation electricity is needed	- Fast response<br>- Activation electricity is needed	- High energy efficiency<br>- Activation electricity is needed*	- No activation electricity<br>- Seasonal dependent performance*<br>- Narrow range of transmittance change	- No activation electricity<br>- Colored in yellow<br>- Fabrication cost	- Activation electricity is needed<br>- Low outside visibility when off-state	- Various color range<br>- Multi-pixel structure<br>- Need precise film thickness control	- Fast response<br>- Use electrothermal energy or surface plasmon effect<br>- Rigid substrate<br>- Limited transmittance changes within infrared wavelength	- A common material<br>- No activation electricity<br>- Transparent at heater-off state<br>- Slow recovery time*

**Note**			*When turn it on/off	*Darker in the winter than in the summer	*Phase transition temperature of pure VO_2 is 68 ^oC #A special situation that an artificial solar lamp (Solax, 500W) was in front of the TC window †An estimation value		*Depends on the initial film thickness	*Only infrared range	*Depends on the heating performance

- **The measurement of standard mechanical stimulus**

The details for the experiment to measure the impact energy of the mechanical stimulus as follow:

The home-made impact generation system which consisted of a syringe tip and a spring created a mechanical stimulus. The stored energy within the spring(s) was released to the chamber (of MTC device) to produce large pressure difference around the syringe tip. This sharp pressure gradient wave eventually led to formation of a seed of crystallization for sodium acetate. Note that a diameter of the syringe tip was 0.5 mm and the injection rate of the syringe tip was approximately 3 mm/sec.

The mechanical stimulus can be adjusted by designing various spring systems, i.e., the different stored energy. The established conditions were:

1. The phase change of the Sodium acetate can be activated by changing the spring constant only (by changing the number of springs in our experiment) while the other conditions are fixed.

2. A probability of a seed creation is strongly correlated to the mechanical impact including spring constant or the stored energy.

We have carried out simple experiments, which show a relationship between an input energy and a seed creation probability. (**Table S2** and **Figure S1(a) & S1(b)**) Note that 20-time-experiment was carried out for each energy condition.

Table S2. Experiment conditions and results regarding mechanical stimulus and crystallization.

Case	1	2	3	4	5	6	7
Applied Mechanical Stimulation (mJ)	6.39	9.68	13.9	26.0	35.5	39.2	48.0
Number of Crystallization Occurrence (Out of 20)	0	0	0	1	3	18	19
Initial compression (cm)	0.21	0.38	0.89	0.9	3.05	3.85	4.3
Input compression (cm)	1	1	1	1	1	1	1
Spring constant (N/cm)	0.9	1.1	1	2	1	0.9	1

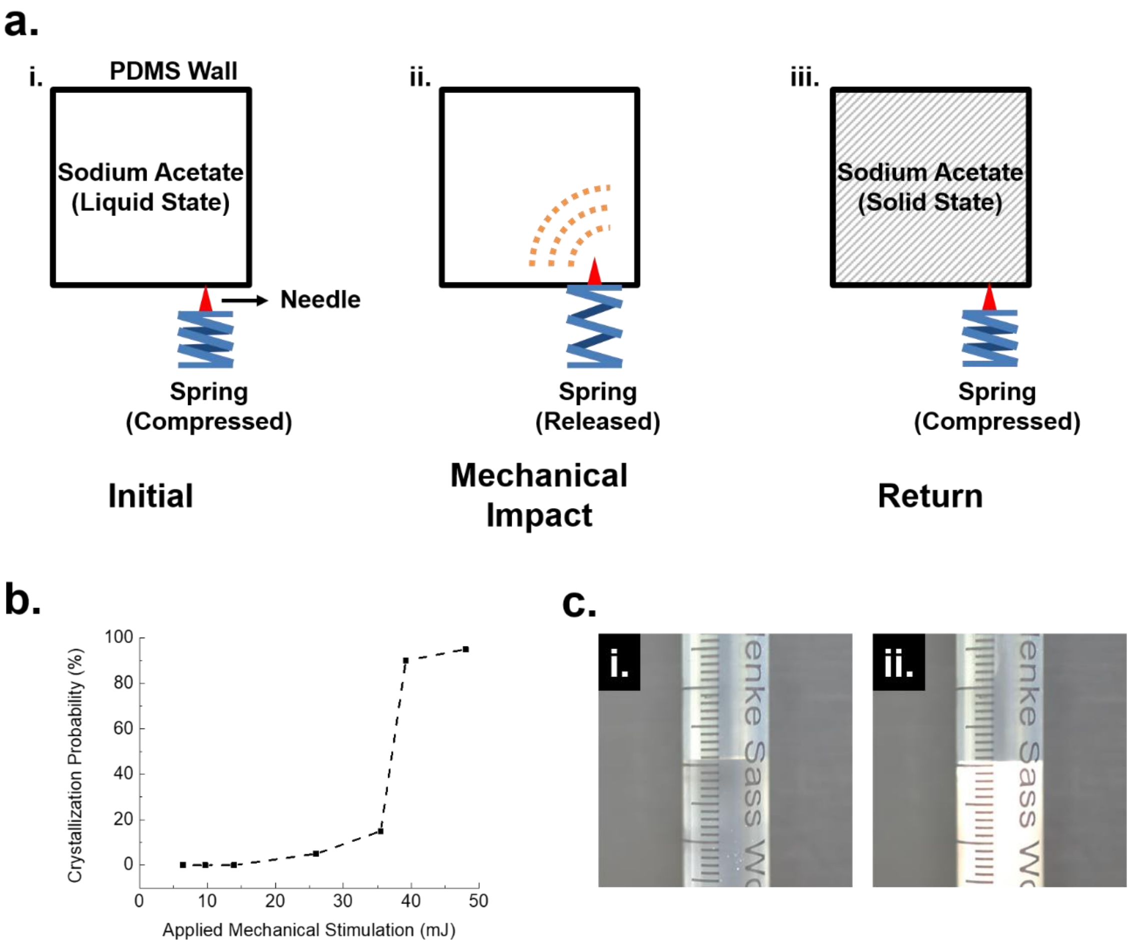

Fig. S1. Mechanical stimulus schematic, crystallization probability, and volume change for crystallization. (**a**) Schematic model for a movement of syringe tip. Note that stored energy in the spring makes large mechanical impact instantly. A movement of the syringe tip was confined between 1st and 2nd hanger. (**b**) Crystallization probability was gradually increased in accordance to increase in applied mechanical power intensity. The standard mechanical stimulus was around 40 mJ. (**c**) Minor volume change was observed (i) before and (ii) after sodium acetate crystallization.

Table S3. Digital images for the sodium acetate crystallization regarding two cases of applied mechanical stimulation. (Photo Credit: Hyunmin Cho & Jinhyeong Kwon, Seoul National University).

**Time**	**Case 1**	**Case 7**
0 sec	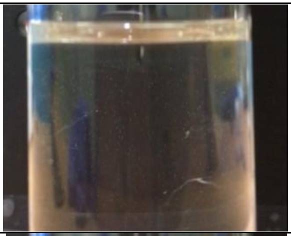	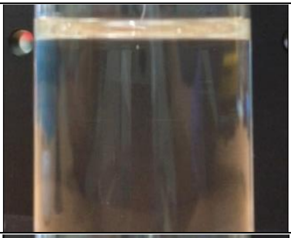
Impact	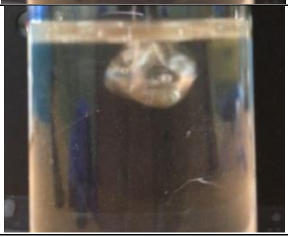	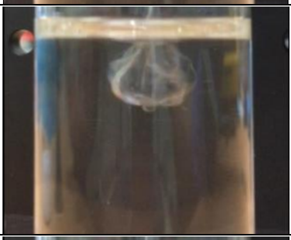
0.5 sec	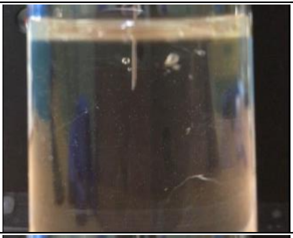	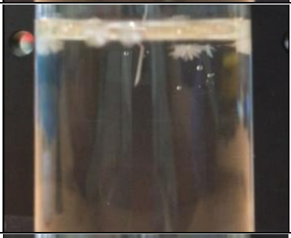
1.0 sec	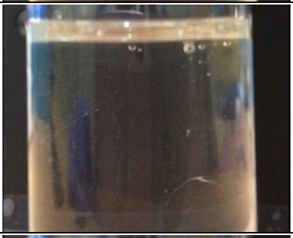	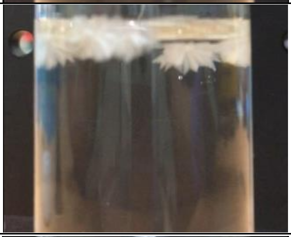
1.5 sec	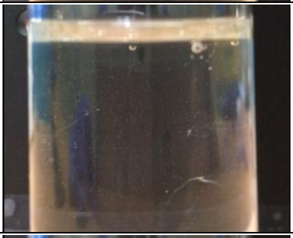	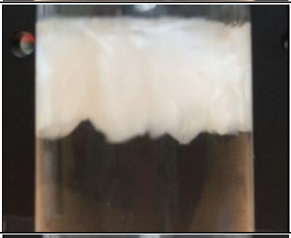
End	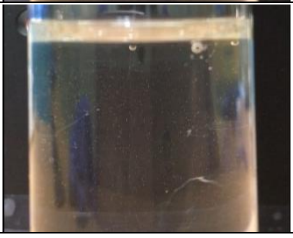	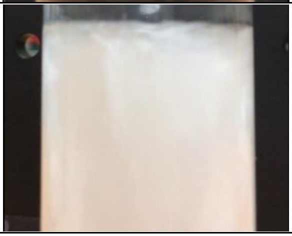

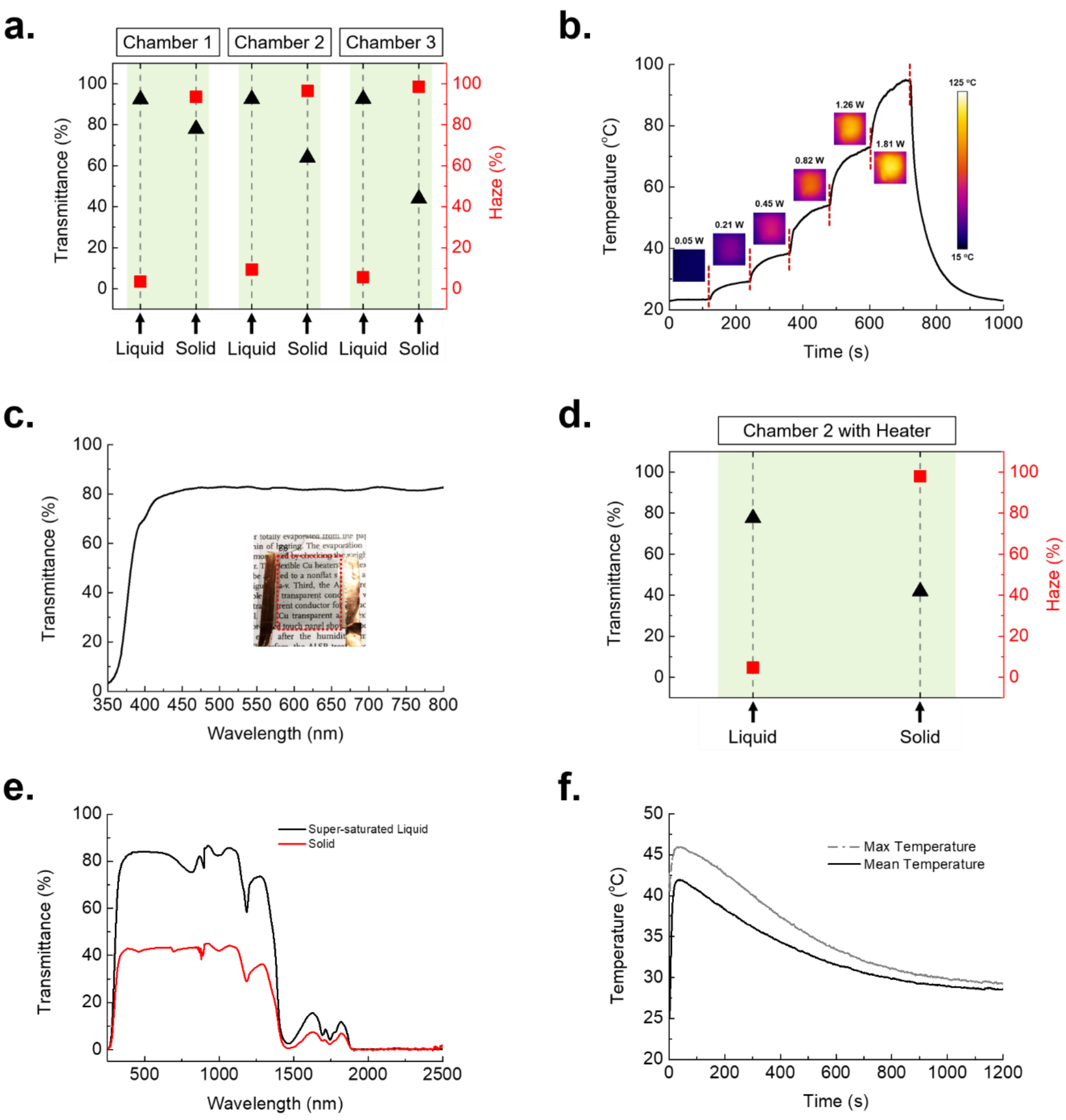

Fig. S2. The optical and thermal behavior analysis of MTC device and microheater. (**a**) The transmittance and haze variation with the sodium acetate phase change as liquid and solid for each chamber. (**b**) The heating performance characterization of the transparent micro heater fabricated by acid-assisted laser sintering process. (**c**) UV-Vis analysis of the micro heater processed by laser and UV-curable epoxy resin. (**d**) The transmittance and haze variation with the sodium acetate phase change for MTC device integrated with the 2 mm thickness chamber and the transparent micro heater. (**e**) The optical transmittance of the MTC device from wavelength of 250 – 2,500 nm. The crystalized (solid) state sodium acetate in MTC device can effectively block UV and visible light as well as IR wavelength region. (**f**) The overall temperature evolution during the crystallization of sodium acetate by the exothermal reaction.

■ **The thermodynamic properties of sodium acetate for exothermal reaction**

The thermodynamic properties of sodium acetate as the following table. In this circumstance, specific heat and convective heat transfer coefficient can be reasonably assumed to be constant, according to their temperature dependent data.

The lumped heat model was applied to the MTC device by following equation

$$m\,C_p \frac{dT}{dt} = \dot{Q} \; - hA(T - T_\infty) \qquad \text{(S1)}$$

Table S4. Various factors and conditions for the calculation of exothermic heat generation.

Chamber width	25 mm
Chamber length	25 mm
Chamber thickness	2 mm
Convective heat transfer coefficient	20 W/m^2·K
Ambient temperature	25 ^{o}C
Latent heat of solidification	225 kJ/kg
Specific heat of sodium acetate	2.024 kJ/kg·K
Specific heat of water	4.2 kJ/kg·K
Density of sodium acetate	1,450 kg/m^3
Density of water	1,000 kg/m^3
Weight percent sodium acetate to water	58%

Note that the heat capacity of the chamber, [kJ/K], was calculated by weight percent of sodium acetate in deionized water. Heat generation rate by solidification was assumed to be linear to the crystal growth (0-6 seconds) and constant until full solidification (6-20 seconds). This assumption was obtained from an experimental temperature profile.

The lumped heat equation, which is ordinary and inhomogeneous linear differential equation, can be solved and the temperature profile of chamber can be derived by using aforementioned conditions and assumptions.

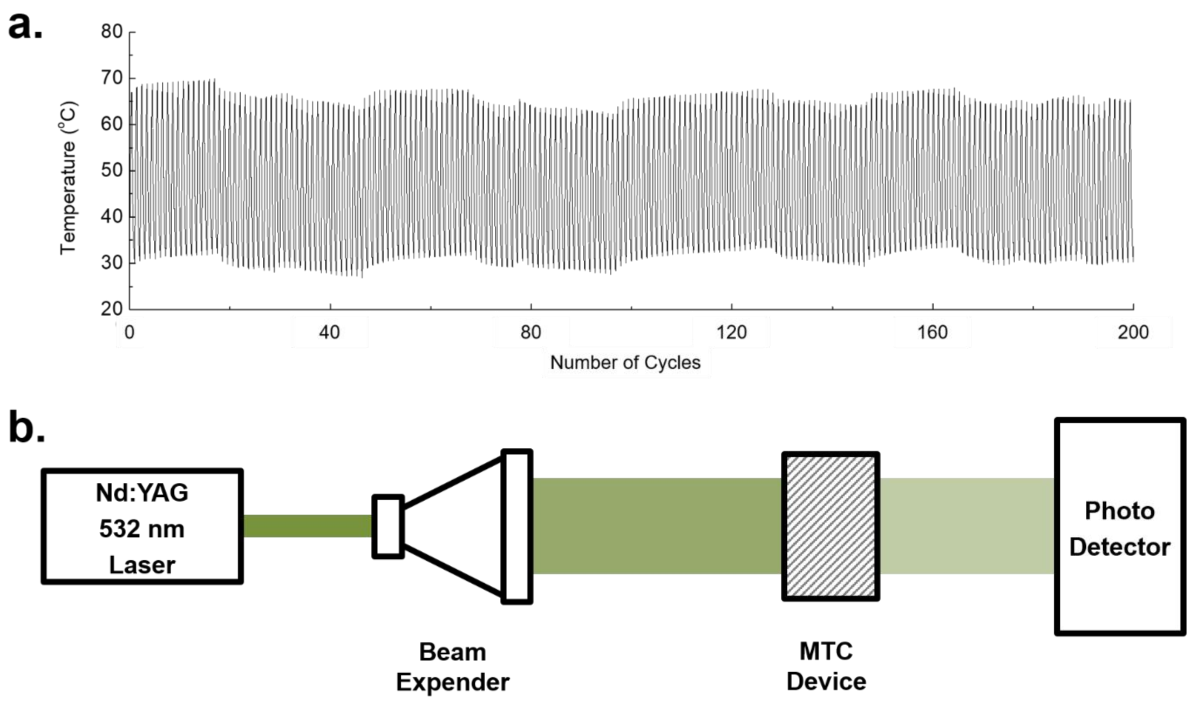

Fig. S3. Cyclic durability test and real-time measure system of transmittance. (**a**) The cyclic test up to 200 cycles shows the durability of MTC device without significant degradation. (**b**) The lab-made optical setup for verifying the transmitted light intensity variation with the phase of MTC device.

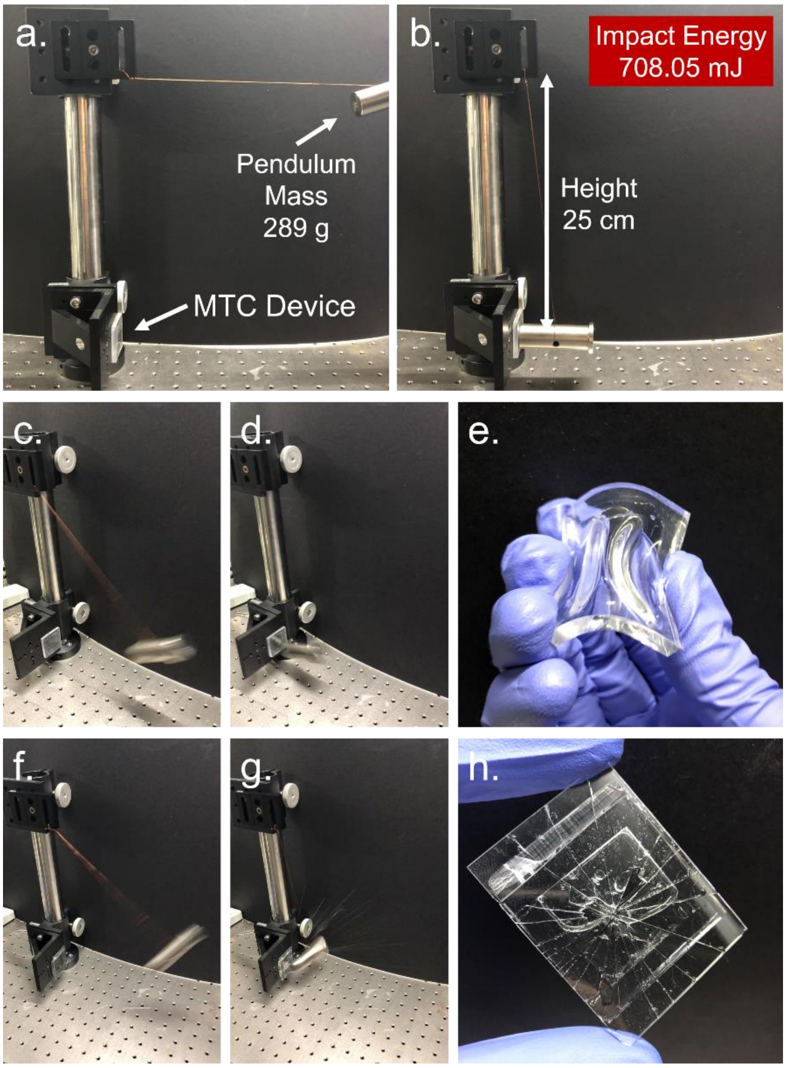

Fig. S4. A pendulum impact test for mechanical external stimulus. The test conditions were represented in a-b. The calculated mechanical impact energy of the pendulum was approximately 708.05 mJ. A sodium acetate solution containing PDMS chamber, the MTC device, showed flawless appearance during and after the test in c-d. The MTC device was stable within external (unexpected) impact and even it beards high flexible state in e. A sodium acetate solution containing glass (surface) chamber was damaged after the same pendulum test in f-g. While the glass was broken by external impact, the sodium acetate solution stayed in solution state shown in h. (Photo Credit: Hyunmin Cho & Jinhyeong Kwon, Seoul National University).

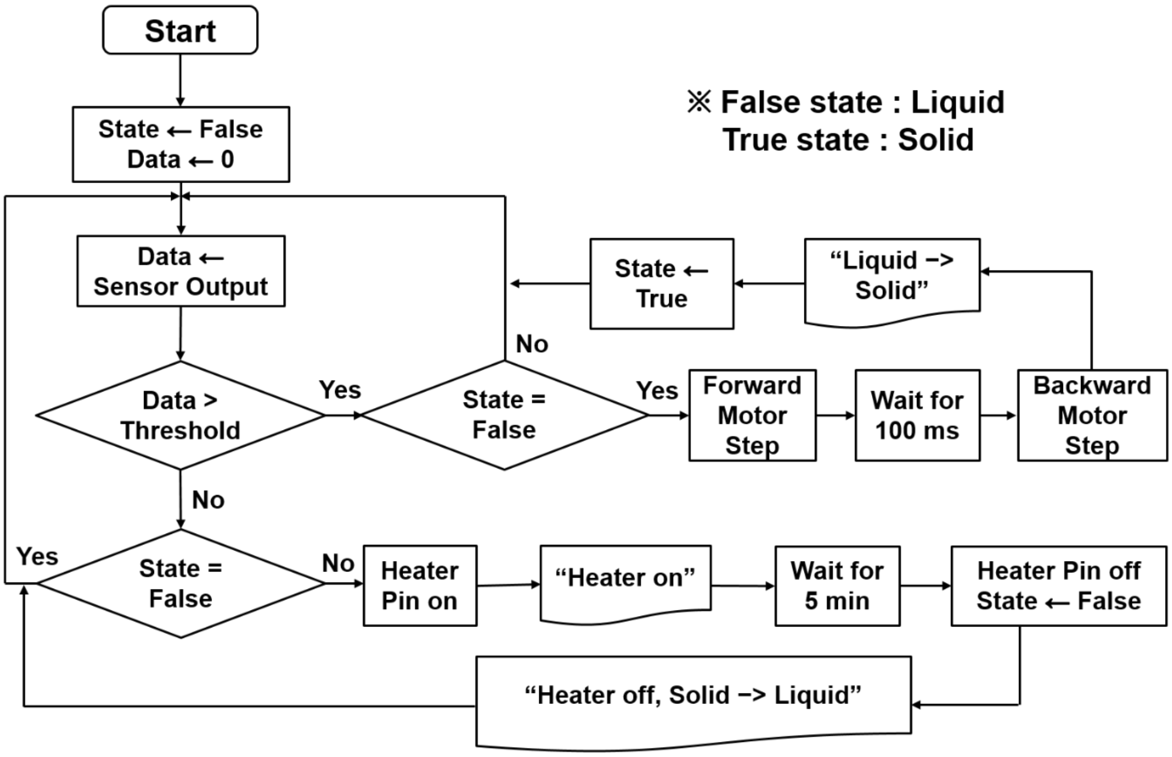

Fig. S5. The logic flow chart for smart glass system integrated with MTC device and control units.

Video Clips

Movie S1. The real-time operation of smart glass system by sodium acetate crystallization with mechanical perturbation when the UV sensor detected UV light.

Movie S2. The real-time operation of smart glass system by sodium acetate phase change to saturated liquid state. Under the critical level of UV intensity, the transparent micro heater turns on to warm up and make the MTC device transparent.
